# Medical Items and News of Interest to the Physician

**Published:** 1872-10

**Authors:** 


**For the Use of Physicians.**

CULLED FROM THE STANDARD MEDICAL JOURNALS OF THE WORLD.

NOTE.—These formulæ are intended only for the regular practitioner of medicine, and should be employed only by him, or under his immediate supervision.

**Tic-douloureux.**

Dr. Bartholow, Cincinnati, Ohio, (*The Clinic*), reported a case of epileptiform tic-douloureux of six years duration, cured by the hypodermic injection of morphia and the iodide of potassium.

**Iodoform as a Neurotic.**

Ephraim Cutter, of Boston, (*Jour. Gynæcological Soc.*), relates cases of uterine diseases, in which the use of iodoform afforded relief as a neurotic.

**Removal of Plaster of Paris Splints.**

This may be easily accomplished by wetting them with a strong solution of common salt.—It causes the plaster to crumble so that the bandage can be easily cut.

**For Otitis.**

The following is recommended by Dr. Brownrigg of Mississippi as a remedy in acute otitis:

Take of Tobacco, ʒj. (cut fine.)
Glycerine, ℥j. M.
S. Five drops in the ear once a day.

**Glycerine of Tar.**

The following is from the same authority:—

Take of Tar, 150 parts.
Yellow of egg, 150 parts.
Glycerine, 300 parts.

Mix. This preparation is of the consistency of a pomade. It does not adhere to the skin, and may be diluted with water.

**Cerebro-Spinal Meningitis.**

Dr. N. S. Davis, Chicago, (*Medical Examiner*), uses with success in this disease the calabar bean, either alone or in combination with ergot. He believes that such remedial agents as have power to diminish excitability, and at the same time increase the vascular tonicity, exert the most favorable influence over the active stages of its progress. Such are the calabar bean, cannabis indica, gelseminum, ergot, etc. In the active stage of the disease he has not found either opiates or quinine to produce any favorable effects.

**Sick Headache.**

Dr. John Murray, of London, (*Lancet*), has had under his care at Middlesex Hospital, cases of sick headache of an hereditary character, which were much benefitted by ten minim doses of indian hemp, three times daily, between the attacks.

---

**Hæmorrhoidal Suppository.**

Dr. Henry M. Field, Newton, Mass., (*Jour. Gynælogical Soc.*), recommends this formula for a suppository in hæmorrhoidal cases:

℞
Acid tannic., gr. v;
Ext. belladon., gr. ss—j;
Butyr. cacao, q. s.

---

**Eczema.**

A correspondent writes us that during the past year he has used, in several cases of chronic eczema, with very much satisfaction to his patients and himself, a solution of chloral as a topical remedy, in the proportion of one or two drachms to a pint of water, applied two or three times a day.—*Boston Med. and Surg. Jour.*

---

**Chronic Rheumatism.**

W. C. Kemp, L. R. C. P., London, writes to the *British Medical Journal*, recounting the cure of numerous cases of chronic rheumatism through the administration of kerosene oil. A teaspoonful was administered in a wineglass of water, every other night. The kerosene produced no unpleasant symptom, no loss of appetite, and no effect upon the bowels or kidneys.

---

**Nævus Maternus.**

Mezgar reports, in *Langenbeck's Archives* a case of telangiectasia cured in the following manner: While the vein leading from the part was compressed with one hand, the vessels were ruptured by a sudden blow from the other.—This was repeated several times, with the result of producing a complete cure without a cicatrix.—*Phila. Med. Times.*

---

**Pneumonia and Pleurisy.**

S. S. Herrick, M. D., (*Richmond and Louisville Med. Jour.*), has prepared a table of twenty-four cases of pneumonia and pleuro-pneumonia, with only three deaths, in which early blistering was resorted to. The beneficial effects were attributed chiefly to the promotion of absorption of effused products through osmosis, by increasing the flow of blood on the stimulated surfaces, and in the adjacent tissues.

**Carminative Powder for Infants.**

This is a Vienna recipe:—

Take of Fennel seed, 25 grains.
Aniseed, 50 grains.
Powdered sugar, 350 grains.
Powdered opium, 1 grain.

Make into a powder, ten grains of which will contain .02 grains of opium.

---

**Tincture of Gelseminum in Tatanus.**

In the *Baltimore Medical Journal* is recorded a recovery from tetanus, for which gelseminum was administered. The disease lasted seventeen days, during which time twenty ounces of the tincture were taken. During the first twelve days the patient took from half a drachm to two drachms every hour.

---

**For Tapeworm.**

*L'Union Medicale* gives the following as Suvacher's formula:—

Take of Castor oil, 60 grammes (15 ʒ by weight.)

Oil of turpentine, 15 grammes (3.8ʒ.)

Gum arabic, 15 grammes (3.8ʒ.)

Mint water, 60 grammes (15 ʒ.)

Simple water 30 grammes (7.6ʒ.)

Mix, and make a potion, to be taken early in the morning at one draught.

---

**Pruritus Vulvæ.**

Dr. McGrath, (*Canada Lancet*), recommends the following lotion in pruritus vulvæ; it should be applied by means of a soft sponge, after ablution, morning and evening:

℞

Biborate of soda, ʒij;

Hydrochlorate of morphia, gr. xx;

Hydrocyanic acid, ʒj;

Glycerine, ℥j;

Distilled rose water, ℥viij. M.

---

**Scarlatina.**

Dr. Chapin, (*Mich. Unv. Med. Jour.*), is very partial to the internal use of carbolic acid, and has come to consider it almost a specific in this disease. He is accustomed to use something like the following formula:

℞

Acid carbolici;

Tr. Opii;

Ætheris cholor., āā ʒj;

Glycerinæ;

Aquæ cinn., āā ℥ij. M.

Sig: A teaspoonful every four hours. He also uses the acid as an application to the throat.

**Emmenagogues.**

Dr. Parvin, (*Am. Practitioner*), gives the subjoined formulæ for emmenagogue pills. In anæmic amenorrhœa, one composed of equal parts of dried sulphate of iron, white turpentine and aloes. The pill may weigh two or three grains, and may be given twice or thrice daily.

Another may be made of equal quantities of rue, savine and ergotine, and half as much either of aloes or of gamboge. The pill may weigh from three to four grains, and two or three may be given three times a day.

---

**Psoas Abscess.**

A prompt cure of psoas abscess following a very large dose of iodide of potash is reported by C. du Hadway, M. D., Jerseyville, Ill., in the *Medical Archives*. The patient, male, German, apparently rebellious to all treatment, was put on the iodide of potash, a solution, ʒij. to aqua ℥vj., of which he was to take a tablespoonful three times daily. Not understanding the directions, the whole one hundred and twenty grains was taken at one dose, and not only did it do him no harm, but his appetite, which before was very poor, was immediately restored, and in ten days his abscess was entirely healed.

---

**Chloroform in the Treatment of Biliary Calculi.**

Dr. John Barclay, in a communication to the *British Medical Journal*, mentions the case of a man who had suffered for twenty-three years from gall-stones, and knowing that ethers are solvents of cholesterine, he prescribed chloroform in doses of two or three drops three or four times a day, on the chance of reaching the calculi through the blood. The pain, tenderness, distension, and jaundice, all disappeared together, and in the eight years which have since elapsed, the patient has never had another attack.

---

**Xylol in Smallpox.**

Dr. Richard Moffett, of Philadelphia, (*Phila. Med. Times*), gave the following prescription to a patient with confluent smallpox, with a happy effect:

℞
Olei xylol, gtt., cc.:
Pulv. accaiæ, q. s.;
Syrp. simple.;
Aquæ, āā ℥j. M.

Sig: A teaspoonful every two hours. He has tried this remedy in a number of cases since, and its use has always been attended with good results.

**Cholera Remedy.**

*New Remedies* contains the following cholera prescription, a favorite one of Dr. H. Hartshorne, of Philadelphia:

℞

Chloroform;

Tinct. opium;

Spts. camphor.

Spts. ammonia aromat., āā f. ʒiss;

Creosote, gtt. iij;

Ol. of Cinnamon, gtt. viij;

Brandy, f. ʒij. Mix.

Dilute a teaspoonful with a wineglass of watter, and give two teaspoonfuls every five minutes, followed by a lump of ice.

---

**To Mask the Taste of Castor Oil.**

According to a correspondent of the Boston *Medical and Surgical Journal*, the following formula affords a method of completely disguising castor oil:—

Glycerine,

Ol. ricini, āā, f℥ij.

Ol. cinnam, ♏iv.

The essential oil should be rubbed up with the glycerine, the castor oil added, and the mixture well shaken before using.

The editor of that excellent quarterly, *New Remedies*, remarks that "This seems to be the best method of disguising castor oil yet devised."

---

**Administration of Chloroform.**

Mr. W. Crooke, M. R. C. S., says in the *Australian Medical Gazette*, that he has discovered a perfectly safe and effectual means for giving chloroform. He describes the process as follows: "Having placed myself in a suitable position, I poured out not more than thirty drops of chloroform on a handkerchief, inhaled gently for a few seconds, then as rapidly as I could.—The sensation of passing into unconsciousness (a most agreeable one,) followed immediately, and I woke up just in time to see the dentist removing a second stump from his forceps. I was perfectly insensible to pain. The whole affair occupied only a few minutes. I felt neither pain, sickness, headache, nor any other inconvenience after it was over." His plan then, is to place but a small quantity of chloroform upon a handkerchief; allows the patient to inhale it slowly for a few seconds, and then quickly and vigorously, until under its influence. He claims that from 30 to 60 drops of chloroform is all that is necessary to produce complete anæsthesia, by this plan.

**Multiple Buboe.**

R. C. Brandeis, M. D., Vienna, correspondent of the *Richmond and Louisville Med. Jour.*, writes that Prof. Zeissl, chief physician for syphilitics in the Vienna Hospital, has achieved much success in the treatment of acute and subacute inguinal and femoral buboes, by the application of a solution of acetate of lead to the glands; and in the case of multiple buboe, the following ointment of iodide of lead will afford speedier relief than the sugar of lead compress:

℞

Plumbi iodati, ʒj:

Ext. belladonnæ, ℈ij;

Emplast. diachyli, ℥j.

Unguent. elemi, q. s. to make soft plaster.

---

**Whooping Cough.**

Mr. John Grantham states in the *British Med. Journal*, that in cases of whooping cough in the last stage, (that is, after the third week,) he has had one ounce of the strongest liquid ammonia put into a gallon of water in an open pan, and the steam kept up by means of halt a brick made red-hot throughout and put into the boiling water containing the ammonia, the pan being placed in the center of the room, into which the patients were brought as the ammoniated steam was passing off. "This method was used in the evening just before bedtime; and it has been so efficacious in abating the spasmodic attack, and after three or four days terminating the malady, that I cannot overestimate the great value of this mode ot inhaling the ammonia as a therapeutic agent in tranquilizing the nervous system in whooping cough."

---

**A Good Diaphoretic.**

Dr. S. C. Osborne, in the *American Practitioner*, highly commends the following formula, stating that in intermittent fever it is of great use, not only in shortening the paroxysm, but also in lessening the dose of quinine necessary to prevent relapse. The first dose usually nauseates for the hour, but after this nausea is absent, and the third dose rarely fails to induce profuse diaphoresis.

℞

Chloroformi

Sp. etheris nitrosi

Tinct. opii camphor

Vini antimonii } . . āā fl ℥ss.

Aquæ . . . . . . . fl ℥vi.

Mix. S. For adults, teaspoonful every hour until the fever abates.

**Cerebro-Spinal Meningitis.**

J. McClay Armstrong, M. D., Edwardsville, Ill., (*Med. and Surg. Reporter*), writes that after the relief of the brain symptoms, spotted fever should be treated as any ordinary case of subacute rheumatism. He uses the following mixtures:

℞
Tinct. xanthoxylum;
Tinct. cimicifuga rac.;
Tinct. guaracum, āā ℥ij;
Tinct. colchicum rad., ℥j;
Pot. acetas, ʒj;
Syr. simplex;
Spts. vini gallici, āā ℥ij. M.

Sig: A dessert spoonful every four hours.

℞

Quin. sul. gr. xj;

Tinct. ferri chl., ʒij;

Syr. simplex;

Aquæ menth. pip., āā ℥j. M.

Sig: A dessert spoonful three times a day, with Dover's powders at night, if necessary.

He has had under his care thirty-two cases of this disease; three proved fatal within forty-eight hours; the remainder recovered. He is also convinced that this disease should be known as cerebro-spinal rheumatosis.

---

**Goitre.**

T. Littell, M. D., of Cincinnati, (*The Clinic*), recently had a patient with goitre, which measured when first seen 21¼ inches in circumference. The treatment was adopted as recommended by Lucke, which consists in the injection into the tumor of the tincture of iodine.—Within three months eleven injections were made into its parenchymia—in form ten to twenty-five drop doses—which reduced the tumor 4 1-5 inches; and its steady and constant decrease was evidence of the efficacy of treatment, and promised future perfect success. The digital compression after removal of the syringe, and the closure of the wound of penetration by adhesive plaster, as recommended by Lucke, were always observed. The suggestion, too, was adopted of permitting the needle to remain in the tumor while the body of the syringe was withdrawn to be refilled, that there might be the least possible danger of injecting the iodine into a minute vein. The almost entire absence of local reaction was probably due to the fact that cold dressings were always applied to the surface after each injection.

**Carbuncles.**

Carbuncles, according to the *Georgia Medical Companion*, are most safely, humanely, and more regularly treated as follows: Introduce the canula of a hypodermic syringe into the center of the tumor, draw out the piston, and with it will come pus, if any be present. The syringe is to be removed from the canula and emptied, the canula left in, and the syringe replaced in the canula again, and the piston withdrawn as before, as long as pus follows. When all the pus is out, withdraw the canula and apply on the tumor, externally, with a brush, as follows:

℞

Collodion, ʒj;

Castor oil, gtts. xx;

Carbolic acid, grs. v;

Tannin, ℈j. M.

Several applications are to be made, one after another, so that a good outer covering is obtained at once. If the patient is weak, give him tr. iron, 20 drops, every four hours; also,

℞
Fl. ext. Peruvian bark, gtt. xx;
Spts. ammon. arom., grs. xx;
Infus. gentian, ℥j. M.

Repeat as often as deemed advisable.

---

**Bronchitis and Catarrhal Pneumonia.**

In obstinate acute bronchitis, after the first intense stage; in catarrhal pneumonia, both of children and adults; in bronchorrœa, and also in ordinary chronic bronchitis, Dr. H. C. Wood, Jr., Phila., (*New Remedies*), has obtained more apparent good from the use of muriate of ammonia than any other remedy. The best formula, he says, is as follows:

Ammoniæ muriat., ʒij;

Ext. glycyrrhiz., ʒj;

Mucl. acaciæ,

Aquæ, āā f. ℥iij. M.

Sig: Tablespoonful for an adult every two hours; teaspoonful for a child, a year old, every three hours.

When patients object to the mixture of sweet and salt, the following is to be preferred:

℞

Ammoniæ muriat, ʒij;

Aquæ, f. ℥vj. M.

Dose as before.

When the cough is very annoying, 1-20 of a grain of sul. of morphia, or ten to fifteen minims of tincture of hyoscyamus, may be added to each dose.

In bronchorrœa the following may, at the same time, be used by inhalation twice or thrice daily:

℞

Sat. solution alum, ʒvj;

Tr. hyoscyamus, ℥ss. M.

---

**Dyspepsia.**

J. E. Nichols, M. D., Osage, Iowa, (*Chicago Med. Jour.*), states that in this affection his main reliance is nux vomica in small tonic doses. If there is little or no trouble beyond the stomach, he gives:

℞

Fluid ext. nux vom., ʒiij;

Alcohol, ʒvj. M.

Sig: Five drops in a little water, before each meal.

If there is some irregularity of the bowels, with the pain of epigastrium around to the right hypochondrium, he combines the mandrake as follows:

℞
Fluid ext. nux vom.;
Fluid ex. mandrake;
Alcohol, āā ʒiij. M.

Sig: As the other.

Where there is much complaint of wakefulness at night, horrid dreams, etc., he gives in addition to the foregoing:

℞

Iodide potassium, ʒss;

Chlorate potassium;

Carbonate potassium, āā ʒj. M.

Fiat pulvis div. in chart. No. xx.

Sig: One in half a glass of warm milk each night at bed time.

If, as is often the case witnessed, pain in the head, throbbing in the temples, he administers bromide of potassium and syrup for a morning potion, as soon as the patient awakes. Sometimes gelseminum is combined thus:

℞
Fluid ext. nux vom.;
Fluid ext. mandrake;
Fluid ext. gelseminum, āā ʒiij. M.

Sig: As the others.

In cases of troublesome nausea, heartburn, acrid or fetid eructations, after eating, he uses phosphate of lime rubbed up with loaf sugar; or, in place of this:

℞
Syrup amanti, ℥vj;
Aromat. sul. acid, ʒij. M.

Sig: A teaspoonful an hour after each meal, or when the eructations are troublesome.

**Epithelioma.**

A noteworthy case is reported by Dr. Fornè, in the *Montpelier Medical*, of last February, showing the excellent effect of topical applications of creosote in epithelioma. A sailor, 24 years of age, presented himself with an ulcer upon the cutaneous border of his upper lip, about four centimetres in diameter. The tissues were perceptibly indurated in its vicinity, there was severe itching pain, and there was no trace of syphilis. After two days observation the ulcer was increasing, and was pronounced epithelioma. Treatment was commenced by dipping a brush in pure creosote, and having removed any that might drop, the whole surface of the ulcer was lightly but firmly touched with the brush, a certain degree of pressure being used and the brush retained on the same point a few seconds so as to insure a thorough application. A piece of lint, moistened in a gummy solution of creosote, was then laid upon the ulcer. The application caused but slight pain. Three days subsequently it was found that the ulcer had not increased. The application was repeated in the same manner, and five times afterward at about the same interval. Cicatrization then commenced and proceeded rapidly, being favored by a dressing of thin paper moistened with a solution of creosote. Entire recovery took place about six weeks after the treatment commenced. This method deserves to be tried in similar cases.

---

**Cyanosis from Nitrate of Silver.**

Dr. L. P. Yandell, Jr., Professor of Clinical Medicine in the University of Louisville, says in the *American Practitioner*, that the discoloration of the skin caused from the administration of nitrate of silver for epilepsy, can be removed by the use of iodide of potassium. He cites the following cases: "The two cases which have suggested this report are similar in many respects. Both were young merchants, and both had been treated unsuccessfully for epilepsy by nitrate of silver in their youth. Both contracted syphilis, and for tertiry symptoms got iodide of potassium. This drug was given in from ten to sixty grain doses thrice daily, for a number of months, in connection with ferruginous or bitter tonics. One of the patients was forced to discontinue the iodide because of its disagree able effect upon the system. The other took it until all traces of syphilis had passed away, and he increased in flesh under its use. In both cases the fading of the stains was gradual. In the first case there is a faint trace of discoloration remaining, though it is scarcely perceptible. In the second, which was the darker of the two, there is not a shadow of the disfigurement. The iodide of potassium was not given in either case in reference to the cyanosis, and its beneficial effects were observed by me accidentally more than a year after their occurrence. It may be well to state that both patients were treated by the moist mercurial vapor bath during much of the time they were using the iodide of potassium, and the abundant diaphorisis may have assisted the action of the iodide. I would suggest, therefore, for the treatment of nitrate of silver cyanopathia, the use of the vapor bath in connection with iodide of potassium."

---

**Pneumonia.**

Dr. Strohl, (*The Practitioner* from *Le Journal de Medicine*), whilst admitting that pneumonia undergoes spontaneous cure in the greater number of cases, thinks that appropriate treatment should be adopted to aid the powers of nature. For many years he has had recourse to the ordinary method of bleeding and the administration of tartar emetic, but since the year 1841, he has been in the habit of giving sugar of lead, which he considers to be the best of internal remedies for this disease. It is preferable, he remarks, to tartar emetic, to digitalis, and to veratra, because its action is more certain, more prompt, and more free from inconvenience. Its action is incontestably superior in the pneumonia of old people. About five grains may be taken per diem in solution, in divided doses.—M. Strohl has never observed the slightest indication of saturnine poisoning in the course of this treatment, and M. Lendet, who has also used it largely, makes the same observation.—Far from producing diarrhœa, it rather occasions constipation. It does not interfere with any of the phenomena concomitant to the critical resolution, as expectoration, diaphoresis, etc. Under its action the pulse rapidly diminishes in frequency, the febrile symptoms disappear, and the temperature falls in the course of six days. The use of lead may be intermitted as soon as the fever has abated and resolution has fairly set in.

---

**Healing of Ulcers by New Method.**

Dr. James Braithwaite, editor of *Braithwaite's Retrospect*, writes to the *British Med. Joural:*

The application of an aqueous solution of carbolic acid (one drachm to eight ounces of water)

to an ill conditioned ulcer, cleans it from all purulent matter, and causes it to assume a healthy red appearance, with each granulation distinctly visible. If a wound in this state be freely exposed to warm, dry air for some hours, it becomes glazed and dried on the surface. It becomes covered by what is practically an impervious transparent membrane, closely applied to the surface of the granulations, and exercising a certain amount of mechanical pressure upon them. In many cases no matter forms underneath this membrane, and cicatrazation goes on underneath it with great rapidity. In time, the membrane assumes the appearance of a thin dry scab, and drops off. If matter forms under the membrane, it is at once visible through it; the lotion should then be re-applied, and the wound dried by exposure, as before. Immediately this is done, the inflamed edges of the ulcer commences to pale in color, and in 24 to 34 hours have nearly the tint of natural skin.—This treatment is especially of value in the cure of ulcers on the leg, as no confinement to bed is necessary; and what is required in the application of the lotion and subsequent drying can be done in the evening, after the conclusion of the day's work. The first case in which I tried this plan was that of Godfrey S. He had been thrown off his ordinary business twice, for a period of four to six weeks, with an ulcer on the shin of the right leg. It healed the first time; but on the second occasion it resisted all treatment, although he laid in bed two weeks. I then tried the plan recommended, and he returned to work in a week. Of course, the wound was unhealed, but it was covered with a firm scale. This dropped off many weeks afterward, leaving a healthy cicatrized surface.—Another case was that of John M., a hair-dresser, accustomed to be on his feet all day. The lower half of his left leg anteriorly was one mass of inflammation and deep ulcers, secreting unhealthy pus, and with irregular edges. This was cured by this plan alone, thanks to his own intelligence and care, without his being one day off his work. I have tried this plan in numerous cases of small wounds, but do not think it applicable for sores of large size—say six or seven inches across—from a scald. I have met with one case that defied this plan of treatment. It was a chilblain on the foot of a very strumous child, just returned from India. She had at the same time pemphigus on the back of both hands. The profuse suppuration was too much for the mechanical pressure of the membrane to check it. And although cleaned, dried and glazed repeatedly, pus always formed underneath. I at last simply dressed it with spermaceti ointment, and it healed by contraction in about two months. Of course, the membrane or scab acts not merely by pressure but by exclusion of the air.

